# Case Report: *SLC52A2* variants cause Brown-Vialetto-Van Laere syndrome type 2, characterized by pure red cell aplastic anemia: clinical and genetic features of three Chinese children

**DOI:** 10.3389/fped.2026.1725574

**Published:** 2026-02-05

**Authors:** Zhenzhen Chen, Landi Lai, Xiaomei Lu, Bomao Zhong, Qi Peng, Ziqiang Liu

**Affiliations:** 1 Child Healthcare Department, Dongguan Children’s Hospital, Dongguan, Guangdong, China; 2 Children’s Health Care Department, Dongguan Gaobu Hospital, Dongguan, Guangdong, China; 3 Laboratory Department, Dongguan Children’s Hospital, Dongguan, Guangdong, China; 4 Department of Medical and Molecular Genetics, Dongguan Institute of Pediatrics, Dongguan, Guangdong, China; 5 Key Laboratory for Children’s Genetics and Infectious Diseases of Dongguan, Dongguan, Guangdong, China

**Keywords:** Brown-Vialetto-Van Laere syndrome type 2, neurodegenerative disease, pure red cell aplasia, riboflavin transporter deficiency, riboflavin treatment, *SLC52A2* gene variant

## Abstract

**Objective:**

To report three Chinese pediatric cases of Brown-Vialetto-Van Laere syndrome type 2 (BVVLS2) presenting with pure red cell aplasia (PRCA) as the core manifestation, and to analyze their clinical features, molecular basis, and response to riboflavin therapy.

**Methods:**

We conducted a retrospective analysis of three pediatric cases, integrating detailed clinical phenotyping with comprehensive genetic analysis (including whole-exome/targeted sequencing, Sanger validation, and ACMG-based variant interpretation). To elucidate genotype-phenotype correlations, we interpreted these findings in the context of a literature review.

**Results:**

All three patients carried compound heterozygous variants in the *SLC52A2* gene. Each exhibited early-onset PRCA (onset age: 2 days to 6 months; hemoglobin: 29–67 g/L) and progressive neurodegeneration, including motor regression, axonal peripheral neuropathy, and sensorineural hearing loss. Riboflavin supplementation led to normalization of hemoglobin levels within four weeks and marked improvement in neurological function.

**Conclusion:**

This case series provides detailed longitudinal data on riboflavin-responsive PRCA as a core presenting feature of BVVLS2 and reports rare *SLC52A2* variants in the Chinese population. Early riboflavin treatment effectively reversed anemia and partially improved neurological deficits, which may inform a new diagnostic and therapeutic approach for unexplained PRCA accompanied by neurodegenerative features.

## Introduction

Brown-Vialetto-Van Laere syndrome (BVVLS), also known as riboflavin transporter deficiency (RTD; OMIM #614707/#211530), is an autosomal recessive disorder caused by pathogenic variants in both alleles of the *SLC52A2* (encoding riboflavin transporter 2, RFVT2) or *SLC52A3* (encoding riboflavin transporter 3, RFVT3) genes. The core pathological mechanism involves impaired transmembrane riboflavin transport, resulting in deficient synthesis of flavin coenzymes—flavin mononucleotide (FMN) and flavin adenine dinucleotide (FAD). This deficiency leads to neuronal energy metabolism dysfunction and axonal degeneration (1). Characteristic clinical features include progressive bulbar palsy, sensorineural hearing loss, and muscular weakness. Approximately 100 cases have been reported worldwide, with an estimated incidence of 1 in 1 million. However, due to phenotypic heterogeneity and frequent misdiagnosis in early stages, the true disease burden is likely substantially underestimated (2).

Riboflavin (Vitamin B₂) is a water-soluble vitamin that cannot be synthesized endogenously and must be obtained through intestinal absorption. In its active forms, FMN and FAD, it serves as an essential cofactor for mitochondrial electron transport chain complexes I and II, as well as multiple redox enzymes. It plays a critical role in biological electron transfer and maintains respiratory chain function, thereby supporting the metabolism of carbohydrates, amino acids, and lipids (3). Brown-Vialetto-Van Laere Syndrome type 2 (BVVLS2) is specifically associated with variants in the *SLC52A2* gene, which cause reduced expression of riboflavin transporter proteins. This impairment leads to diminished cellular riboflavin uptake, declined FMN/FAD synthesis, and subsequent mitochondrial dysfunction, oxidative stress, and neuronal axonal degeneration—particularly affecting cranial and motor neurons. Common clinical manifestations include gait disturbance, ataxia, optic atrophy, hearing loss, and muscle weakness, often progressing to fatal respiratory failure (4, 5).

Advances in genomic research have revealed that many hereditary neurological disorders may co-occur with systemic involvement. Notably, BVVLS2 presenting with pure red cell aplasia (PRCA) as a dominant feature is exceedingly rare. PRCA is a form of anemia characterized by severe impairment of erythropoiesis in the bone marrow. Traditional etiologies include congenital disorders, autoimmune dysfunction, viral infections, and thymoma (6). While hereditary forms of PRCA—such as Diamond-Blackfan anemia—have garnered increasing attention, the mechanism underlying *SLC52A2*-related PRCA remains poorly understood.

In this study, we define “riboflavin-responsive PRCA” as a severe normocytic anemia characterized by selective erythroid hypoplasia in the bone marrow, which shows rapid and complete hematological remission upon riboflavin supplementation. While a recent study described riboflavin-responsive erythroid hypoplasia in BVVLS2 (7), in our cohort, severe, transfusion-dependent PRCA was the earliest and dominant manifestation in two of three patients, preceding overt neurological decline in two of three patients. Here, we report three Chinese children with compound heterozygous *SLC52A2* variants, in whom early-onset anemia and neurodegeneration were reversed by riboflavin, underscoring its vital role in hematopoiesis. These findings establish a new diagnostic paradigm for unexplained pediatric PRCA with neurological features.

## Patients and methods

This study was approved by the Institutional Review Board of the Children's Hospital and conducted in accordance with the study protocol and the principles of the Declaration of Helsinki. Written informed consent was obtained from the parents of all participants.

### Patients

The clinical and genetic characteristics of the three patients are summarized in Table 1.

**Table 1:** Clinical and genetic characteristics and treatment response of three pediatric patients with BVVLS2 and PRCA.

Characteristic	Case 1	Case 2	Case 3
Sex/Age at report	Male, 4 years	Male, 1 year 11 months	Male, 1 year
Age at anemia onset	20 days	2 days	6 months
Key neurological features	Motor regression (18 months), visual pathway damage, tongue fasciculations, axonal neuropathy	Motor regression (7–8 months), auditory pathway damage, mixed neuropathy	Motor stagnation (6 months), swallowing difficulties, visual/auditory pathway damage, tongue fasciculations, axonal neuropathy
*SLC52A2 (NM_001363118.2)* Variant			
Variant 1 (cDNA; protein)	c.1135_1137del (p.Trp379del)	c.588C>G (p.Phe196Leu)	c.1247C>T (p.Ser416Phe)
ACMG Classification (Evidence)^a^	VUS	VUS	VUS
	PM2_Supporting, PM4	PM2_Supporting, PM3, PP3_Moderate	PM2_Supporting,PM3, PP3_Moderate
Variant 2 (cDNA; protein)	c.350T>C (p.Leu117Pro)	c.593G>A (p.Trp198*)	c.75_76insCCTGG (p.Ala26Profs*42)
ACMG Classification (Evidence)	VUS	P	LP
	PM2_Supporting, PM3, PP3_Moderate	PVS1_VeryStrong, PM2_Supporting, PM3_Supporting	PVS1, PM2_Supporting
Inheritance pattern	Compound heterozygous (Autosomal recessive)	Compound heterozygous (Autosomal recessive)	Compound heterozygous (Autosomal recessive)
Hematologic parameters (at diagnosis)			
Hemoglobin, min (g/L)	45	29	67
White blood cell count (×10⁹/L)	5.03	8.16	4.80
Platelet count (×10⁹/L)	432	526	544
Reticulocyte count (%)^b^	0.25	0.63	0.93
Response to riboflavin			
Hb after 4 weeks (g/L)	140	130	125

a ACMG variant classification: VUS, variant of uncertain significance; P, pathogenic; LP, likely pathogenic. ^b^Normal reference range for reticulocyte count: 0.5%–1.5%.

### Case 1

**Figure 1:**
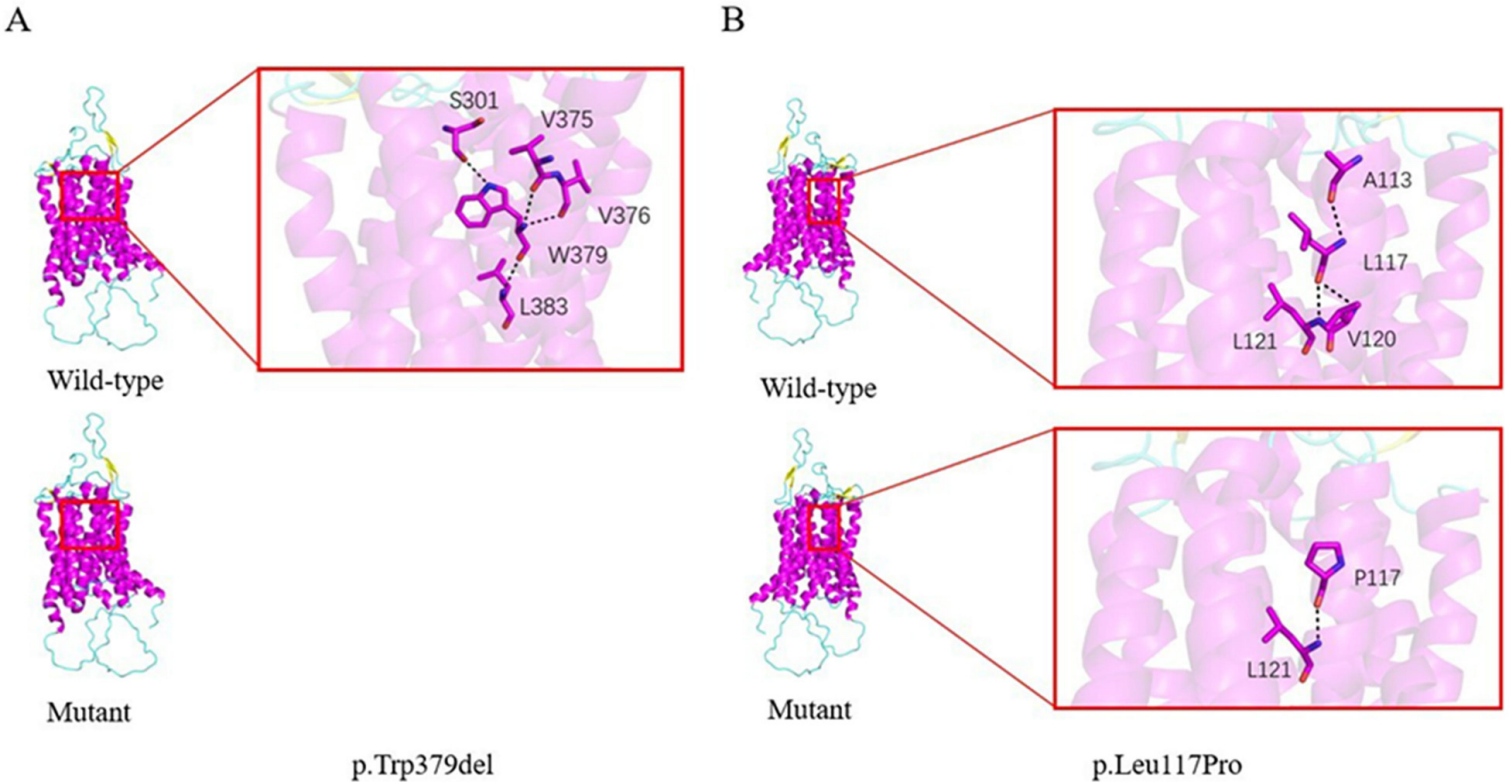
Predicted structural impact of *SLC52A2* variants in case 1. **(A) p.Trp379del:** In the wild-type RFVT2 model, Trp379 forms hydrogen bonds (dashed lines) with surrounding residues (Ser301, Val375, Val376, Leu383), The in-frame deletion of this residue is predicted to disrupt these interactions and destabilize the local protien structure. **(B) p.Leu117Pro:** Substitution of leucine with proline at position 117 is predicted to disrupt the backbone conformation and alter interaction with adjacent residues (Ala113, Val120).

A 4-year-old boy presented with early-onset PRCA, with hemoglobin (Hb) levels as low as 45 g/L beginning at 20 days of age, along with progressive neurodegeneration. His perinatal history was unremarkable. Hematologically, bone marrow examination revealed selective erythroid hypoplasia with otherwise normocellular marrow, and he did not respond to immunosuppressive therapy (prednisone and cyclosporine). Neurologically, he exhibited motor regression starting at 18 months, including loss of sitting stability and eventual inability to stand. Additional findings included axonal peripheral neuropathy (reduced amplitude of common peroneal nerve motor waves on electromyography), visual pathway impairment, scoliosis, and restrictive pulmonary dysfunction. Genetic testing revealed compound heterozygous variants in *SLC52A2*: c.1135_1137delTGG (p.Trp379del) and c.350T>C (p.Leu117Pro) (Figure 1). He was diagnosed with BVVLS2 accompanied by PRCA.

### Case 2

**Figure 2:**
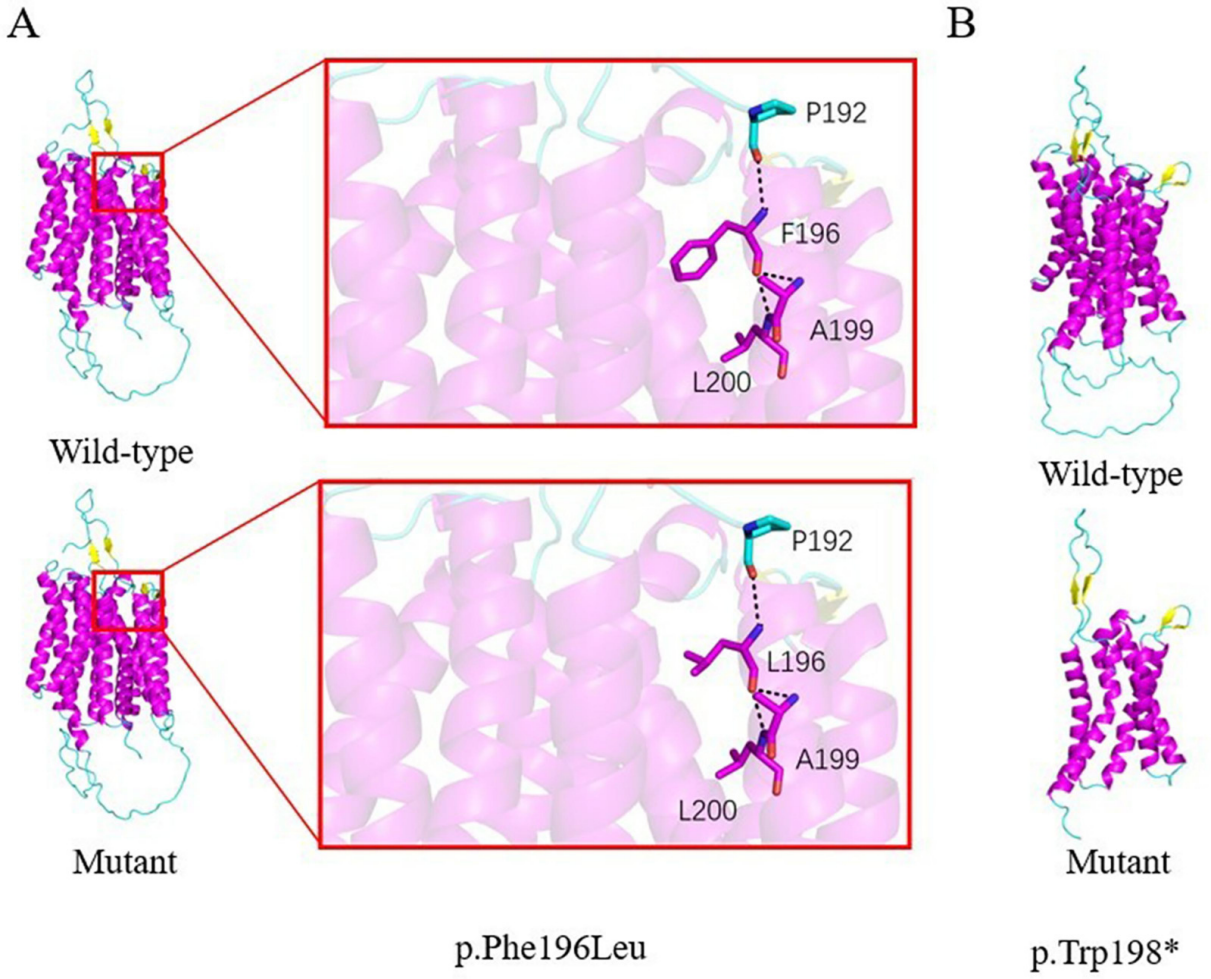
Predicted structural impact of *SLC52A2* variants in case 2. **(A) p.Phe196Leu:** The substitution of phenylalaine with leucine at position 196 is not predicted to alter local residue interactions. However, this change may affect substrate recognition or protein-protein interactions critical for riboflavin trnasport. (B) p.Trp198*: This nonsense variant introduces a premature termination codon, resulting in a severely truncated protein that lacks the complete transmembrane channel structure, thereby abolishing transporty function.

A boy aged 1 year and 11 months had early-onset PRCA with a minimum Hb of 29 g/L from 2 days after birth, alongside progressive neurodegeneration and an unremarkable perinatal history. Bone marrow evaluation revealed selective erythroid hypoplasia with otherwise normocellular marrow, and he remained transfusion-dependent. Neurological manifestations included developmental stagnation by 7 months and regression by 12 months, with loss of independent sitting ability. He also exhibited brainstem involvement (symmetrical T2 hyperintensities in the midbrain and pons on MRI), mixed peripheral neuropathy, sensorineural hearing loss (absent auditory brainstem responses), scoliosis, and restrictive pulmonary dysfunction. Genetic analysis identified compound heterozygous *SLC52A2* variants: c.588C>G (p.Phe196Leu) and c.593G>A (p.Trp198*) (Figure 2). A final diagnosis of BVVLS2 with PRCA was made.

### Case 3

**Figure 3:**
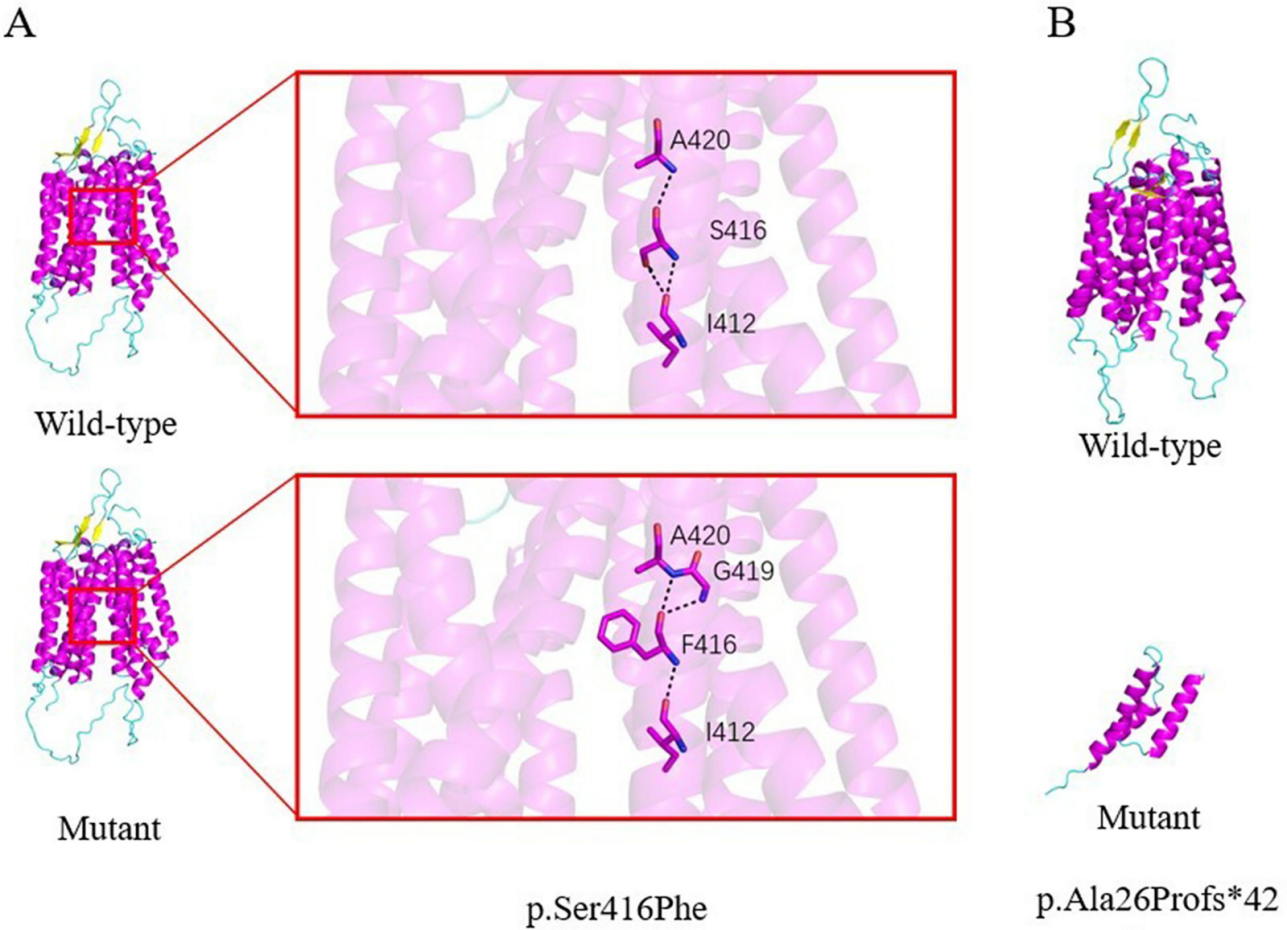
Predicted structural impact of *SLC52A2* variants in case 3. **(A) p.Ser416Phe:** Substitution of serine with phenylalaine at position 416 is predicted to introduce a bulkier hydrophobic side chain, potentially altering local conformation and hydrogen-bonding interactions (e.g., with Gly419). (B) p.Ala26Profs*42: This frameshift variants results in a premature stop codon after residue 26, leading to a severely truncated protein that lacks most of the transmembrane domains and is predicted to be non-functional.

A 1-year-old boy developed PRCA at 6 months of age (Hb 67 g/L) and showed progressive neurodegeneration, with no perinatal abnormalities. Bone marrow stimulation revealed selective erythroid hypoplasia with otherwise normocellular marrow. Neurologically, motor stagnation and swallowing difficulties emerged at 6 months, followed by widespread axonal peripheral neuropathy and visual/auditory pathway damage (abnormal ABR and VEP). Genetic testing confirmed compound heterozygous *SLC52A2* variants: c.1247C>T (p.Ser416Phe) and c.75_76insCCTGG (p.Ala26Profs*42) (Figure 3). He was diagnosed with BVVLS2 and PRCA.

### Genetic testing and analysis

Genomic DNA was extracted from peripheral blood samples obtained from all probands and their parents after informed consent. Due to the distinct clinical presentations and diagnostic pathways of each case, different high-throughput sequencing strategies were applied.

For Case 1, who presented with predominant early-onset anemia followed by motor regression, the initial diagnostic suspicion focused on a broad spectrum of neuromuscular disorders. Therefore, targeted next-generation sequencing (NGS) using a custom panel encompassing 927 genes associated with neuromuscular diseases (My Genostics, Beijing, China) was performed on the proband. Library preparation and sequencing were conducted following the manufacturer's protocols on an Illumina HiSeq 2500 platform. Candidate variants identified were confirmed by Sanger sequencing in the proband. Segregation analysis was then performed in the family, including unaffected parents and sibling.

For Cases 2 and 3, where the combination of severe infantile anemia and rapid neurodegenerative decline suggested a broader genetic etiology beyond neuromuscular disorders, trio-based whole-exome sequencing (WES) was performed. Sequencing was carried out on an Illumina NovaSeq 6000 platform, achieving a mean coverage depth of >100× across targeted regions, with >95% of bases covered at ≥20×.

### Bioinformatic processing and variant analysis

For both the targeted panel and WES data, sequence reads were aligned to the GRCh37/hg19 reference genome using BWA-MEM. Variant calling followed GATK best practices. Identified single-nucleotide variants (SNVs) and small insertions/deletions (indels) were annotated and filtered using an in-house pipeline integrated with public population and disease databases (e.g., gnomAD, 1000 Genomes, ClinVar, HGMD, OMIM).

### Variant interpretation and pathogenicity assessment

The potential functional impacts of missense variants were evaluated using multiple in silico prediction tools (including PolyPhen-2, SIFT, MutationTaster, and FATHMM-MKL). The evolutionary conservation of affected amino acids was analyzed via multiple sequence alignment. The transmembrane topology of the *SLC52A2* protein was predicted using TMHMM and SOSUI servers to contextualize the variant locations. Final pathogenicity classification for all candidate variants was performed according to the standards and guidelines of the American College of Medical Genetics and Genomics (ACMG).

**Figure 4:**
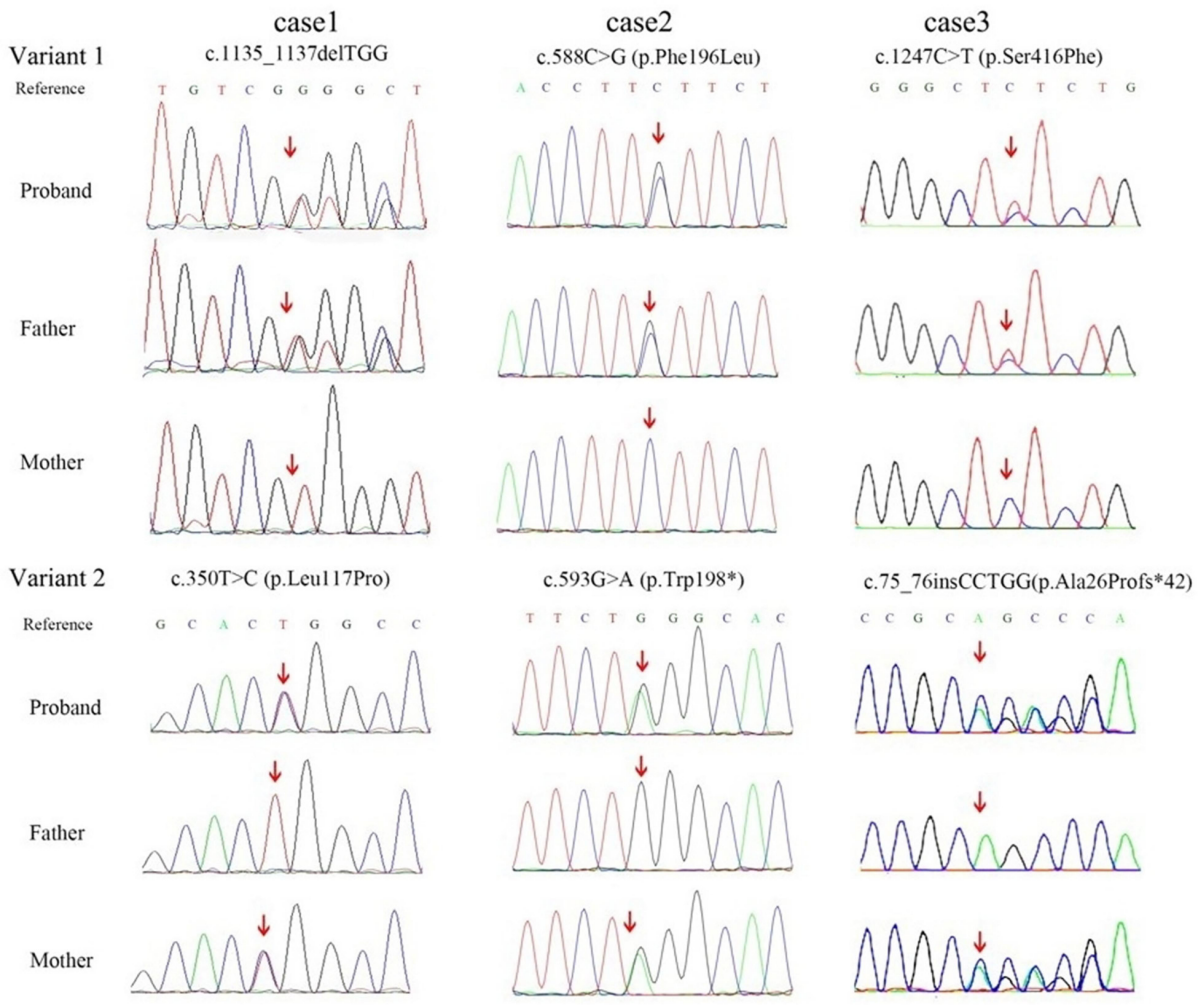
Sanger sequencing chromatograms and segregation analysis *SLC52A2* variants in three families with BVVLS2. For each of the three cases (cases 1–3), the compound heterozygous variants are shown. For each variant, chromatograms rom the proband and both parent are aliened. Arrows indicate the location of the variant nucleotide or nucleotides. The segregation pattern demonstrates biparental inheritance, confirming the autosomal recessive compound heterozygous state.

All variants were confirmed by Sanger sequencing, performed by commercial diagnostic laboratories. Representative chromatograms are provided in Figure 4. These chromatograms, derived from clinical genetic testing reports, demonstrate the variant identifications and segregation patterns within the families.

### Structural modeling and analysis

To assess the structural impact of identified *SLC52A2* variants, a three-dimensional model of the human RFVT2 transporter (UniProt ID: Q9HAB3) was generated using AlphaFold2. The predicted structure was visualized and analyzed using PyMOL. The potential effects of missense variants on protein stability and ligand interaction were evaluated computationally using DynaMut2 for free energy change (ΔΔG) prediction and Missense3D for detecting residue-level structural disruptions.

## Treatment and outcomes

### Case 1

The patient began oral riboflavin at 20 mg/day (∼1.7 mg/kg/day). Hemoglobin levels increased to 117 g/L after 2 weeks and normalized to 140 g/L by 4 weeks. The riboflavin dose was titrated to a maintenance level of 70 mg/day, with complete resolution of anemia. Motor function improved significantly: at 2 months, gross motor gains were noted; by 6 months, his motor function showed marked improvement, progressing to independent walking over short distances, corresponding to a 40% increase in his total Gross Motor Function Measure-88（GMFM-88）score.; at 12 months, gait stabilized; by 18 months, he could run slowly; and after 36 months, he achieved independent stair navigation and returned to preschool.

### Case 2

Riboflavin was initiated at 20 mg/day (∼1.6 mg/kg/day). Hemoglobin normalized to 130 g/L within 4 weeks, with a maintenance dose of 120 mg/day in divided doses. Anemia resolved completely. With adjunct limb rehabilitation, upper limb function improved by 2 months (active grasping returned), and the patient could stand with support. By 6 months, he achieved brief independent standing (≤30 s), and by 12 months, prolonged independent standing was possible. At 18 months, he could take a few supported steps.

### Case 3

Treatment started with riboflavin 20 mg/day (∼3.0 mg/kg/day). Hemoglobin stabilized around 125 g/L after 4 weeks, with a maintenance dose of 70 mg/day and full hematologic recovery. Auditory and visual responsiveness improved within 1 month. By 2 months, swallowing difficulties had eased, though motor progress remained limited (still unable to sit independently).

In summary, all three patients presented with early-onset normocytic PRCA requiring transfusion, followed by progressive motor and sensory neurodegeneration. Hematological response to riboflavin was rapid (within 4 weeks), while neurological improvement was gradual and variant-dependent.

## Discussion

BVVLS2 is an autosomal recessive disorder caused by biallelic pathogenic variants in the *SLC52A2* gene, classically characterized by progressive neurodegeneration (4). This study reports three Chinese pediatric cases that expand the phenotypic spectrum of BVVLS2, by identifying severe, riboflavin-responsive PRCA as a core presenting feature. All patients presented with early-onset, PRCA as a dominant and core clinical feature, which demonstrated a rapid and complete hematological response to riboflavin supplementation. Notably, the neurological regression observed in our patients is not an intrinsic feature of PRCA, but rather a core manifestation of the underlying BVVLS2, highlighting the multisystem nature of this riboflavin transporter deficiency.

The mutational spectrum of *SLC52A2* is diverse, predominantly comprising missense variants, followed by nonsense, frameshift, and splice-site alterations. These variants are not randomly distributed but show significant clustering within the transmembrane domains, which are critical for riboflavin transport function (1, 8). Analyses of genotype-phenotype correlations have delineated several key associations. First, the genotypic context significantly impacts disease severity. Compound heterozygosity (particularly when one or both variants are loss-of-function) is consistently associated with a higher risk of respiratory insufficiency (4). In contrast, homozygosity for missense variants more frequently presents with ataxia as a predominant and early feature (1, 4). The founder variant p.Gly306Arg exemplifies this. Homozygosity is strongly linked to prominent ataxia, whereas its presence in compound heterozygosity substantially increases the risk of severe respiratory insufficiency (4, 9). Second, the location of variants may influence the clinical phenotype. Based on limited data, variants situated within transmembrane domains appear to be associated with a higher frequency of specific sensory impairments, such as optic atrophy (4). Furthermore, specific combinations of variants—such as combinations of missense variants or transmembrane and extramembrane variants—appear to confer a heightened risk of respiratory complications (4). Despite these advances in correlating genotypes with neurological outcomes, systematic hematological involvement—such as PRCA—remains a genetically uncharacterized and underreported aspect of the BVVLS2 phenotype (7, 10).

Genetic analysis in our cohort identified six different *SLC52A2* variants (p.Trp379del, p.Leu117Pro, p.Phe196Leu, p.Trp198*, p.Ser416Phe, p.Ala26Profs*42), and the variants were associated with the severe PRCA phenotype. These variants included those located in regions less commonly involved: p.Ala26Profs*42 is located in the N-terminus, and p.Ser416Phe is in the extracellular C-terminal tail. Prior functional studies have established that the N-terminal domain is critical for RFVT2 membrane localization and stability (11). The p.Ala26Profs*42 truncating variant is expected to lead to a complete loss of function. The severe neurological impairment in Patient 3 may be explained by this predicted null allele in combination with the p.Ser416Phe variant.

Our study establishes severe, riboflavin-responsive PRCA as a major and treatable systemic manifestation of BVVLS2. The consistent hematological phenotype across patients with diverse variants—truncating and missense—indicates that prominent hematological manifestations are not strictly associated with any single variant type or domain. Rather, these manifestations likely represent a severe, systemic metabolic consequence of riboflavin deficiency that can be revealed by various loss-of-function mechanisms. This finding expands the phenotypic spectrum of BVVLS2 beyond a purely neuro-centric view and underscores the critical role of riboflavin transport in erythropoiesis.

This prominent hematopoietic involvement raises a key mechanistic question: why is erythropoiesis particularly susceptible to riboflavin transporter deficiency? The convergence of severe PRCA across various *SLC52A2* variants suggests a shared, cell-intrinsic defect. We propose that this susceptibility arises from the following factors.

Cell-Autonomous Defect in Erythropoiesis. RFVT2 is highly expressed in erythroid progenitor cells (12). The identified variants, including those classified as variants of uncertain significance (VUS), are predicted to impair riboflavin transport, which would be expected to result in intracellular deficiency of the active cofactors FMN and FAD. Based on the known pathophysiology of riboflavin deficiency, such a deficit could trigger a cascade of metabolic disturbances potentially detrimental to erythroid precursors: (i) potential impairment mitochondrial complex I (FAD-dependent) function, causing an energy crisis (13); (ii) likely reduction in the activity of FAD-dependent glutathione reductase, exacerbating oxidative stress and promoting apoptosis (14); and (iii) possible disruption of iron metabolism, further hampering hemoglobin synthesis (15). This triad of mechanistic perturbations selectively targets the riboflavin-demanding process of erythropoiesis, a mechanism supported by *SLC52A2* knockout mouse models that develop severe anemia with erythroid progenitor depletion (12). Recent clinical reports of BVVLS2 patients with riboflavin-responsive erythroid hypoplasia and vacuolated erythroid precursors further substantiate this direct cytotoxic effect on the erythroid lineage (7).
Variant-Specific “Metabolic Bottleneck”. All patients in this series presented with normocytic PRCA, contrasting with earlier reports of macrocytic anemia in BVVLS2 (10, 16). We hypothesize that different variant types may impair erythroid metabolism through distinct mechanisms. Truncating variants (e.g., p.Trp198*, p.Ala26Profs*42) likely cause a complete loss of function, leading to a generalized metabolic defect. In contrast, we hypothesize that specific missense variants (e.g., the VUS p.Leu117Pro) might partially preserve transport function while still potentially creating a specific “metabolic bottleneck” that is insufficient to support high-flux riboflavin demands of actively proliferating erythroid precursors. The consistent bone marrow finding of selective erythroid hypoplasia with preserved other lineages provides direct histological evidence for this lineage-specific metabolic vulnerability.
Insights from Treatment Response Heterogeneity. The rapid hematological remission achieved with moderate-dose riboflavin (≤3 mg/kg/day) confirms that bypassing the defective transporter can correct the systemic, including bone marrow, deficiency. The slower and more variable neurological recovery likely reflects the additional challenge of achieving therapeutic riboflavin levels within the central nervous system due to blood-brain barrier constraints (17). Furthermore, the transient response to immunosuppressants in Patient 1, but not in Patient 2, hints at potential variant-dependent differences in immune dysregulation secondary to riboflavin deficiency, which may modulate the PRCA phenotype in some cases.

## Conclusion

In conclusion, this study systematically defines and characterizes riboflavin-responsive PRCA as a prominent and treatable manifestation of BVVLS2, highlighting its importance for accurate diagnosis and effective treatment. Together with recent literature (7), our findings establish pathogenic *SLC52A2* variants as an important, genetically defined cause of hereditary PRCA. Therefore, BVVLS2 should be considered in the differential diagnosis of any unexplained infantile or childhood PRCA, particularly when accompanied by neurological signs. Early genetic testing and/or a therapeutic trial of riboflavin are warranted.

The main limitation of this study is its small sample size. Future work should involve larger cohorts to refine genotype-phenotype correlations. Utilizing patient-derived induced pluripotent stem cells (iPSCs) differentiated into erythroid progenitors could directly validate the proposed “metabolic bottleneck” hypothesis and riboflavin rescue *in vitro*. Finally, developing therapeutic strategies to enhance riboflavin delivery to the central nervous system (e.g., via prodrugs or nanocarriers) may help overcome the current therapeutic plateau and further improve neurological outcomes.

## Data Availability

The original contributions presented in the study are publicly available. This data can be found here: ClinVar database, accession numbers: Case 1: SCV007329763, SCV007334861, Case 2: SCV007329765, SCV007334862 and Case 3: SCV007329771, SCV007334863.
